# 12-Molybdophosphoric acid anchored on aminopropylsilanized magnetic graphene oxide nanosheets (Fe_3_O_4_/GrOSi(CH_2_)_3_–NH_2_/H_3_PMo_12_O_40_): a novel magnetically recoverable solid catalyst for H_2_O_2_-mediated oxidation of benzylic alcohols under solvent-free conditions

**DOI:** 10.1039/c8ra00312b

**Published:** 2018-02-12

**Authors:** Saeed Farhadi, Mohammad Hakimi, Mansoureh Maleki

**Affiliations:** Chemistry Department, Lorestan University Khorramabad 68151-44316 Iran sfarhadi1348@yahoo.com +98-6633120618 +98-6633120611; Chemistry Department, Payame Noor University Tehran 19395-4697 Iran

## Abstract

In this work, 12-molybdophosphoric acid (H_3_PMo_12_O_40_, HPMo) was chemically anchored onto the surface of aminosilanized magnetic graphene oxide (Fe_3_O_4_/GrOSi(CH_2_)_3_–NH_2_) and was characterized using different physicochemical techniques, such as powder X-ray diffraction (XRD), Fourier transform infrared spectroscopy (FT-IR), Raman spectroscopy, energy-dispersive X-ray analysis (EDX), scanning electron microscopy (SEM), BET specific surface area analysis and magnetic measurements. The results demonstrated the successful loading of HPMo (∼31.5 wt%) on the surface of magnetic aminosilanized graphene oxide. XRD patterns, N_2_ adsorption–desorption isotherms and SEM images confirm the mesostructure of the sample. FT-IR and EDX spectra indicate the presence of the PMo_12_O_40_^3−^ polyanions in the nanocomposite. The as-prepared Fe_3_O_4_/GrOSi(CH_2_)_3_–NH_2_/HPMo nanocomposite has a specific surface area of 76.36 m^2^ g^−1^ that is much higher than that of pure HPMo. The selective oxidation of benzyl alcohol to benzaldehyde was initially studied as a benchmark reaction to evaluate the catalytic performance of the Fe_3_O_4_/GrOSi(CH_2_)_3_–NH_2_/HPMo catalyst. Then, the oxidation of a variety of substituted primary and secondary activated benzylic alcohols was evaluated with H_2_O_2_ under solvent-free conditions. Under the optimized conditions, all alcohols were converted into the corresponding aldehydes and ketones with very high selectivity (≥99%) in moderate to excellent yields (60–96%). The high catalytic performance of the nanocomposite was ascribed to its higher specific surface area and more efficient electron transfer, probably due to the presence of GrO nanosheets. The nanocomposite catalyst is readily recovered from the reaction mixture by a usual magnet and reused at least four times without any observable change in structure and catalytic activity.

## Abstract 2

12-Molybdophosphoric acid was anchored on magnetic aminopropylsilanized graphene oxide nanosheets and used as a magnetically recoverable catalyst for solvent-free selective oxidation of benzylic alcohols into the carbonyl compounds with H_2_O_2_.

## Graphical abstract

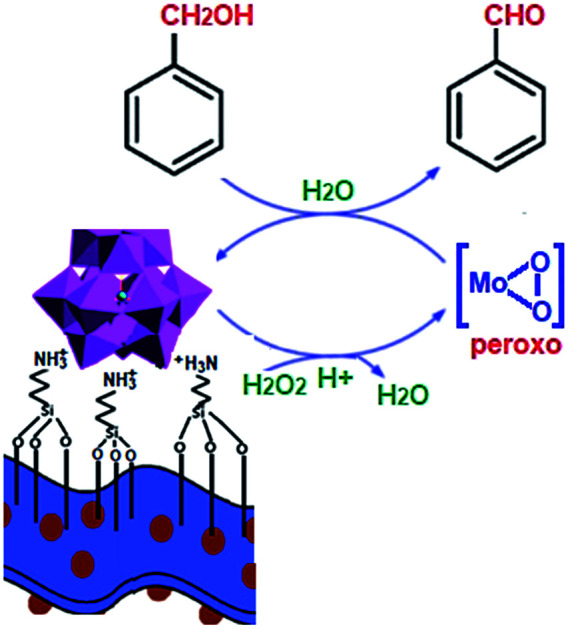

## 1. Introduction

The oxidation of alcohols to the corresponding carbonyl compounds, *i.e.*, benzyl alcohol to benzaldehyde, is one of the most important and vital transformations in synthetic organic chemistry.^1^ Typically, the chemoselective oxidation of alcohols to aldehydes or ketones without traceable over-oxidation products (*i.e.*, carboxylic acids) is attractive for the preparation of fine chemicals owing to the nature of aldehydes or ketones as an intermediate for producing carbonyl products, which are of great importance and versatility for the chemical industry and pharmaceutical synthesis.^2^ Numerous efforts on developing efficient catalysts towards these oxidations have been progressed and reported.^3^ Solvent-free approach is an attractive green process for selective oxidation of benzyl alcohol and oxidants like molecular oxygen, hydrogen peroxide (H_2_O_2_) and TBHP are in the order as green options.^4^ Molecular oxygen as oxidant is not very active, while H_2_O_2_ is an active oxygen donor in catalytic oxygen-transfer reactions. However, most of the catalyst systems using H_2_O_2_ are based on noble metals such as Pt, Pd, and Ru which are expensive and difficult to synthesize.^5–7^ From the practical and environmental points of view, there is a strong demand for the screening out efficient catalytic systems using inexpensive and environmentally benign metal catalysts and non-toxic H_2_O_2_ or O_2_ as the sole terminal oxidant. In this context, polyoxometalates (POMs) are definitely an attractive alternative in terms of economic viability and easy-to manufacture alternative with a heterogeneous nature.

Polyoxometalates (POMs) are a large family of bulky clusters of transition metal oxide anions with structural diversity.^8^ Among various POMs, Keggin-type heteropoly acids (*e.g.* H_3_PMo_12_O_40_ and H_3_PW_12_O_40_) have received much attention and numerous organic transformations can be catalyzed by them, not only due to their controllable and reversible multielectron redox and acidic properties, but also due to their environmentally benign behavior.^9,10^ Moreover, these compounds have moderately high thermal stability in solid state, relatively simple synthesis procedure, and ability to form pseudo-liquid phases.^11^ In despite of the above advantages, there exist two major drawbacks in the catalytic systems involving pristine POMs: (i) low surface area in nonpolar solvents (<10 m^2^ g^−1^) hindering accessibility of reactants to active sites and (ii) high solubility in polar solvents producing recovery and reuse problems. To overcome these obstacles, many researchers have tried to design heterogeneous catalysts by incorporating HPAs into the structure of solid supports such as SiO_2_,^12,13^ Al_2_O_3_,^14^ activated carbon,^15^ TiO_2_,^16^ zeolites,^17^ organic materials,^18,19^ clays,^20^ ZrO_2_–CeO_2_ (ref. 21) and metal–organic frameworks (MOFs).^22,23^ However, most of these supports have some limitations, such as low loading of POM, high leaching of POM especially in polar medium and/or active sites that are unevenly dispersed. Therefore, finding suitable solid supports to overcome the drawbacks is important. Supports modified with functional groups such as carboxylic groups, lactam, amide, imide or amino-groups can allow solving this problem.^24,25^ Especially, the amino-modified metal oxides supports are commonly used for immobilizing POMs not only because of the exceptionally high stability and surface area but also because the amount and the basicity of anchored functional amino groups can be expected to be important in determining the guest–host interactions of the materials.^26–30^ This approach allows obtaining high dispersion of the POMs with minimal leaching. Despite facile recovery, such heterogeneous POM catalysts often suffer from the poor accessibility of the H_2_O_2_ during the oxidation of alcohols with aqueous H_2_O_2_ due to the hydrophobicity of support. Hence, enhancing the accessibility of the oxidant is crucial to the heterogeneous catalyst in the alcohols oxidation with H_2_O_2_.

Among various carbon-based nanomaterials, graphene oxide (GrO) has been proven as an effective support for the immobilization of inorganic and organic materials owing to its large theoretical specific surface area (∼2630 m^2^ g^−1^) and the presence of numerous oxygen containing functional groups on its surface.^31–39^ Due to these rich functional groups, GrO can be easily reacted with organic molecules to generate stable chemically functionalized GrO.^40^ In addition, in comparison with other carbonaceous nanomaterials, GrO may be more environmental friendly and have better biocompatibility.^41^ However, it is difficult to separate GrO from aqueous solutions using traditional filtration and centrifugation methods during and after the process due to its hydrophilic nature and small particle size which increase the cost of industrial application.^42^ The magnetic separation method is considered as a rapid and effective technique for separating nanomaterials from aqueous solution.^43–45^ Hence, magnetic graphene-based composites with large specific surface area and magnetic separation have begun to be used in the field of organic transformations.^46–50^

On the basis of the above discussions, in this work, amino-organosilane functionalized magnetic graphene oxide (Fe_3_O_4_/GrOSi(CH_2_)_3_–NH_2_) was synthesized by a facile method and used as a novel magnetic GrO-based support. Due to relatively high surface area and porosity, insolubility in water and easy magnetically separation, the Fe_3_O_4_/GrOSi(CH_2_)_3_–NH_2_ is an appropriate solid support to anchor Keggin-type PMo_12_O_40_^3−^ polyanion. The ternary magnetic nanocomposite material (abbreviated as Fe_3_O_4_/GrOSi(CH_2_)_3_–NH_2_/HPMo) was prepared by a simple acid–base electrostatic interaction between H_3_PMo_12_O_40_ and amino groups of the Fe_3_O_4_/GrOSi(CH_2_)_3_–NH_2_ support. The coupling of PMo_12_O_40_^3−^ anion with Fe_3_O_4_/GrOSi(CH_2_)_3_–NH_2_ could improve the surface area and avoid the dissolution of HPMo. This novel magnetically recyclable heterogeneous catalyst was used for selective oxidation of alcohols with H_2_O_2_ as a green oxidant under solvent free conditions. Our catalysts showed high catalytic performance in H_2_O_2_-mediated alcohol oxidations under solvent-free conditions. The resulting Fe_3_O_4_/GrOSi(CH_2_)_3_–NH_2_/HPMo composite could be used as a magnetically separable and efficient catalyst for alcohol oxidation under solvent free conditions.

## 2. Experimental

### 2.1 Materials

12-Molybdophosphoric acid (H_3_PMo_12_O_40_, 98%), graphite powder (C, 99.95%) and 3-aminopropyltriethoxysilane (APTES, 99%) were purchased from Merck Chemical Co. All alcohols and other chemicals were commercially purchased and used without further purification.

### 2.2 Preparation of aminosilanized magnetic graphene oxide

Graphene oxide (GrO) was prepared by the modified Hummers method through the oxidation of graphite powder.^51,52^ Briefly, graphite powder (2.0 g) and NaNO_3_ (1.0 g) were mixed with 40 mL of concentrated H_2_SO_4_ in a 500 mL flask and stirrer for 1 hour in an ice bath. Then KMnO_4_ (6.0 g) was added into the vigorously stirred suspension slowly below 15 °C. The ice bath was then removed, and the mixture was stirred at room temperature until it slowly became a brownish slurry, and then it was diluted with 100 mL of water. The reaction temperature was rapidly increased to 98 °C with effervescence, and the color changed to brown. After that, 200 mL of water and 20 mL of H_2_O_2_ (30 wt%) were added. For purification, the mixture was centrifuged and washed with 10% HCl and then deionized water several times to remove the residual metal ions and acid. After centrifuging and drying at room temperature, GrO was obtained as a powder. To prepare Fe_3_O_4_/GrO, 0.25 g of GrO was dispersed in 90 mL water by sonication for 1 hour. Then, 0.84 g of (NH_4_)_2_Fe(SO_4_)_2_ and 2.08 g of (NH_4_)Fe(SO_4_)_2_ were added to the GrO dispersion and its pH was adjusted at 12 by adding 1 mol L^−1^ NaOH. The mixture was stirred at 50 °C for 2 h, filtered and washed with water and ethanol three times. The resulting solid was Fe_3_O/GrO. To prepare amino functionalized magnetic graphene oxide, 0.40 g of the as-synthesized Fe_3_O_4_/GrO dispersed in 50 mL of water, 150 mL ethanol and 5 mL of 3-aminopropyltriethoxysilane (APTES) were added to a round bottom flask. The mixture was stirred for 30 min in room temperature and then was refluxed at about 80 °C for 24 h. After the reaction, the solid was separated by a magnet and washed with ethanol to remove the unreacted APTES. The final product was dried at 80 °C in vacuum for 12 h to obtain the Fe_3_O_4_/GrOSi(CH_2_)_3_–NH_2_.

### 2.3 Preparation of the Fe_3_O_4_/GrOSi(CH_2_)_3_–NH_2_/HPMo nanocomposite catalyst

1 g the as-prepared Fe_3_O_4_/GrOSi(CH_2_)_3_–NH_2_ was dispersed in 80 mL water and sonicated for 1 h. To the above suspension, 1 g HPMo in 200 mL ethanol was added and sonicated for another 1 h. The resulting mixture was stirred at room temperature for 24 h, and then filtered, washed with deionized water and ethanol three times to remove the unreacted HPMo. The final product was dried at 60 °C in open air to obtain magnetic Fe_3_O_4_/GrOSi(CH_2_)_3_–NH_2_/HPMo hybrid nanomaterial. According to the elemental analysis (ICP-AES) results and molecular weight of H_3_PMo_12_O_40_, the loading amount (wt%) of HPMo in Fe_3_O_4_/GrOSi(CH_2_)_3_–NH_2_/HPMo was estimated to be 31.5%.

### 2.4 General procedure for oxidation of benzylic alcohols with H_2_O_2_ over the Fe_3_O_4_/GrOSi(CH_2_)_3_–NH_2_/HPMo nanocomposite

Benzylic alcohol (10 mmol) and catalyst Fe_3_O_4_/GrOSi(CH_2_)_3_–NH_2_/HPMo (0.2 g) were added to a 25 mL flask. Under refluxing conditions, vigorous stirring, and the heating temperature of 100 °C, the aqueous H_2_O_2_ (30 wt%, 15 mmol) was added into the above mixture within 5 min, then the reaction mixture was stirred for 4 h. The progress of the reaction was monitored by TLC and/or GC. After reaction, the solid catalyst was removed by an external magnet, and the liquid was analyzed using a gas chromatography (GC SP-6890) equipped with an FID detector and a capillary column (SE-54; internal diameter = 0.32 mm, length = 30 m) using He as the carrier gas. In the GC experiments, *n*-decane was used as internal standard and the yields were determined by using peak area. The isolated yield was obtained using silica gel plate or column chromatography with a mixture of ethyl acetate/*n*-hexane as an eluent, and the products was characterized by GC-MS. The recovered catalyst for recycling tests was obtained by magnetic separation, washing with ethanol and dried.

### 2.5 Recyclability test of the catalyst

The activity of the recovered catalyst provides useful information about its stability during the catalytic cycle. In order to recover, the magnetic catalyst was separated from the reaction mixture by a magnet and washed three times with distilled water and ethanol. It was further dried at 100 °C for 2 h. The recovered catalyst was then used in the reaction with a fresh reaction mixture and products were analyzed after the reaction.

### 2.6 Characterization techniques

The infrared spectra were recorded at room temperature on a Schimadzu FT-IR 160 spectrophotometer in the 4000–400 cm^−1^ region using KBr pellets. The XRD patterns of powder were recorded on a Rigaku D-max C III X-ray diffractometer using Ni-filtered Cu Kα radiation (*λ* = 1.54184 Å). The morphology of samples was studied by MIRA3 TESCAN scanning electron microscope equipped with energy dispersive X-ray analyzer (EDX) for the elemental analysis. Optical adsorption spectra were obtained from a Cary 100 Varian UV-Vis spectrophotometer in a wavelength range of 200–800 nm. The Brunauer–Emmett–Teller (BET) surface area was measured by N_2_ adsorption measurements at 77 K using a Nova 2000 instrument. The concentration of Mo in the composite was determined by inductively coupled plasma atomic emission spectroscopy (ICP-AES, model OEC-730). The content of HPMo in the nanocomposite was determined by inductively coupled plasma atomic emission spectroscopy (ICP-AES, model OEC-730). Magnetic measurements were carried out at room temperature using a vibrating sample magnetometer (VSM, Magnetic Daneshpajoh Kashan Co., Iran) with a maximum magnetic field of 10 kOe. Raman spectra were obtained using a Raman microscope (SENTERRA-2009, Germany) with Laser wavenumber of 785 nm at 785 nm. Thin-layer chromatography (TLC) was conducted on glass plates coated with silica gel GF254. GC-MS analysis was carried out on a Shimadzu QP 5050 GC-MS instrument with 5973 MS detector at an ionization voltage of 70 eV and equipped with a HP-INNOWAX capillary column (internal diameter = 0.25 mm, length = 30 m).

## 3. Results and discussion

### 3.1 Characterization of the hybrid nanocatalyst

**Fig. 1:**
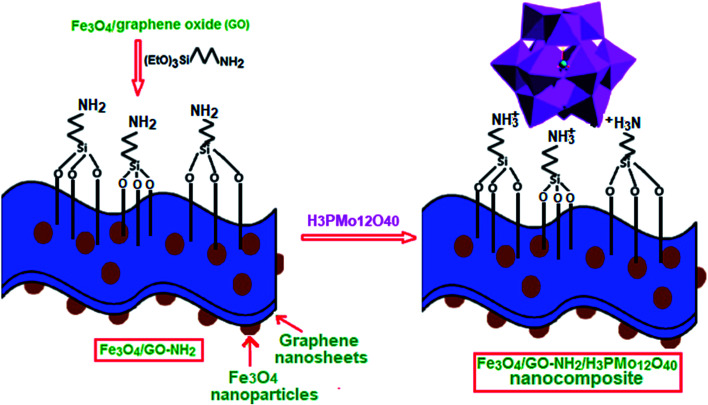
Preparation process of the magnetic Fe_3_O_4_/GrOSi(CH_2_)_3_–NH_2_/HPMo catalyst.

In this work, Keggin-type H_3_PMo_12_O_40_ (HPMo) was chemically immobilized onto 3-aminopropyl functionalized graphene oxide nanosheets decorated with magnetic Fe_3_O_4_ nanoparticles. The preparation process of Fe_3_O_4_/GrOSi(CH_2_)_3_–NH_2_/HPMo hybrid is illustrated in Fig. 1. These suggest that attaching organic bases on GrO might afford an efficient, reusable and environmentally benign base support for heteropoly acid such as HPMo. This novel magnetically recyclable hybrid nanomaterial was constructed by protonating amino groups anchored on the magnetic GrO nanosheets with Keggin-type heteropoly acid HPMo. The surface of GrOSi(CH_2_)_3_–NH_2_ is positively charged by the protonation of –NH_2_ groups by reacting with HPMo, which is benefit for anchoring polyanion *via* an electrostatic interaction. To confirm the successful construction of Fe_3_O_4_/GrOSi(CH_2_)_3_–NH_2_/HPMo hybrid, element analysis was employed. It reveals that the Fe_3_O_4_/GrOSi(CH_2_)_3_–NH_2_/HPMo contains 36.5% Mo, indicating that HPMo was anchored on GrO nanosheets. Along with the anchoring of HPMo on Fe_3_O_4_/GrOSi(CH_2_)_3_–NH_2_, the –NH_2_ groups can be protonated to form the –NH_3_^+^ groups, which absorbs the HPMo polyanions *via* the electrostatic (ionic) attraction interaction.^53^ And due to the electrostatic attraction, the hydrogen bonds between –NH_3_^+^ and HPMo polyanions molecules are reinforced.^54,55^ The structure and composition of the hybrid nanomaterial was further characterized by XRD, FT-IR, Raman spectra, EDX, SEM, VSM and BET surface area analyses.

**Fig. 2:**
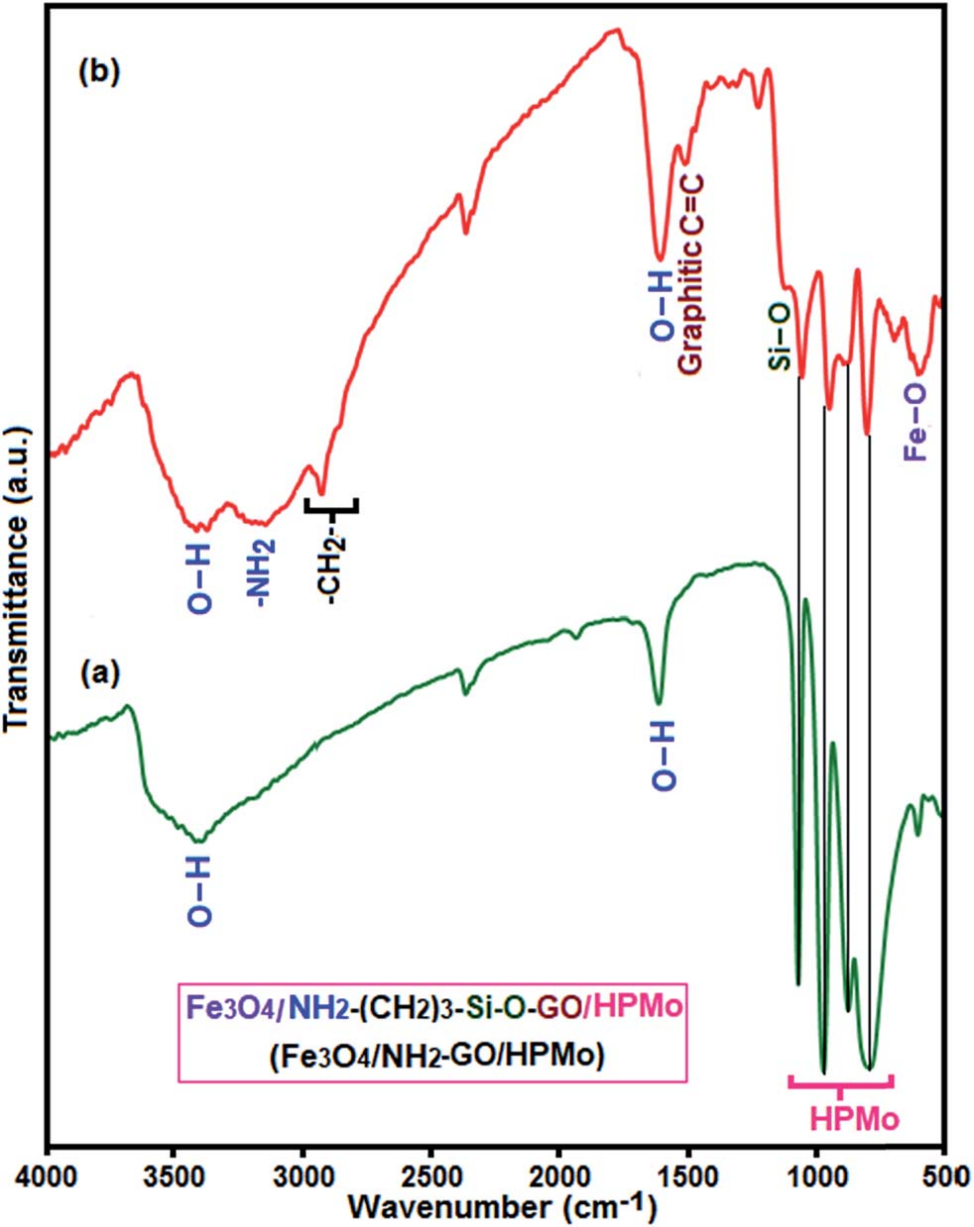
FT-IR spectra of (a) pure HPMo and (d) Fe_3_O_4_/GrOSi(CH_2_)_3_–NH_2_/HPMo.

The FT-IR spectra of synthesized materials are shown in Fig. 2. It is well known that Keggin-type polyoxometalate (PMo_12_O_40_^3−^) contains a cluster of Mo(vi) ions linked by oxygen atoms with a tetrahedral phosphate group. PMo_12_O_40_^3−^ has four characteristic vibration bands as shown in Fig. 2(a): 1066 cm^−1^ for asymmetric stretch vibration of P–O_a_ (O_a_ corresponds to oxygen atom of tetrahedral phosphate group), 966 cm^−1^ for asymmetric stretch vibration of Mo=O_t_ (O_t_ corresponds to the terminal oxygen atoms), 870 cm^−1^ for bending vibration of Mo–O_b_–Mo (O_b_ corresponds to oxygen atom bridging the two tungsten atoms), and 786 cm^−1^ for bending vibration of Mo–O_c_–Mo (O_c_ represents oxygen atom at the corners of the Keggin structure).^56,57^ The FT-IR spectrum of the composite sample is shown in Fig. 2(b). The characteristic absorption bands at 1053, 945, 875 and 798 cm^−1^ corresponding to the P–O_a_, Mo=O_t_, Mo–O_b_–Mo and Mo–O_c_–Mo band vibrations confirm the presence of PMo_12_O_40_^3−^ cluster. The graphitic C=C stretching band at about 1505 cm^−1^ and a strong band at about 588 cm^−1^ assigned to the grapheme nanosheets and Fe–O stretching vibration of the spinel-type Fe_3_O_4_ structure, respectively.^58–60^ And the presence of the anchored APTES chain to the GrO sheets was confirmed by stretching vibrations of C–H bond (2926 and 2850 cm^−1^), the stretching and bending modes of –NH_2_ bonds (3430 and 1630 cm^−1^) and the C–N stretching vibration (1223 cm^−1^).^61^ The Si–O–C stretching vibration (1116 cm^−1^) shows the successful grafting of APTES onto magnetic GrO nanosheet through covalent bonds. All bands appeared the IR spectrum of the hybrid nanomaterial demonstrate the coexistence of PMo_12_O_40_^3−^, Fe_3_O_4_ and GrOSi(CH_2_)_3_–NH_2_ in the hybrid nanomaterial. The shift of some peaks of HPMo in the Fe_3_O_4_/GrOSi(CH_2_)_3_–NH_2_/HPMo compared to the parent HPMo can be attributed to hydrogen bonding and strong electrostatic attraction between negatively charged PMo_12_O_40_^3−^ and positively charged Fe_3_O_4_/GrOSi(CH_2_)_3_–NH_3_^+^ surface.^62,63^

**Fig. 3:**
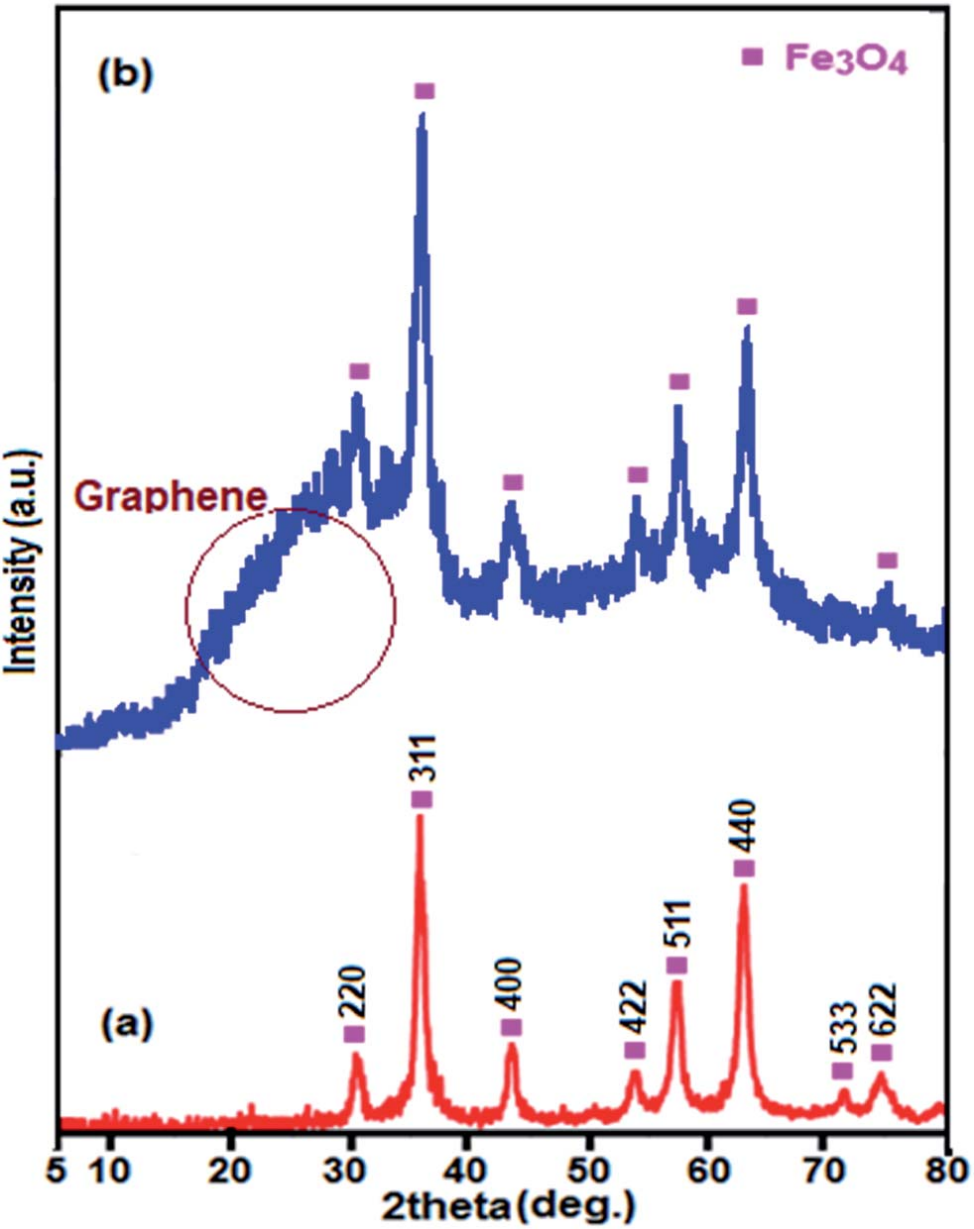
XRD patterns of (a) pure Fe_3_O_4_ and (b) Fe_3_O_4_/GrOSi(CH_2_)_3_–NH_2_/HPMo.

Fig. 3 displays the XRD patterns for Fe_3_O_4_ and Fe_3_O_4_/GrOSi(CH_2_)_3_–NH_2_/HPMo samples. All of the diffraction patterns in Fig. 3(a) and (b) are similar and can be indexed to the Fe_3_O_4_ phase (JCPDS no. 41-1488). No characteristic diffraction peaks of the HPMo appeared which presumably was due to the low content incorporation of HPMo. Also, this result implies that the Keggin unit homogeneously disperses into the GrO nanosheets, which will be benefit to enhance the catalytic activity of the hybrid nanomaterial. Moreover, no characteristic diffraction peaks for GrO are observed in the pattern indicating that the GrO nanosheets do not stack during the synthesis process. The reason can be attributed to that the Fe_3_O_4_ nanoparticles, aminopropyl groups and HPMo anchored on the surfaces of GrO prevent the exfoliated GrO nanosheets from restacking.^64^ However, a broad peak at about 23.5° corresponding to the reduced GrO was appeared, indicating that GrO nanosheets were reduced to graphene during the functionalization process.^65^

**Fig. 4:**
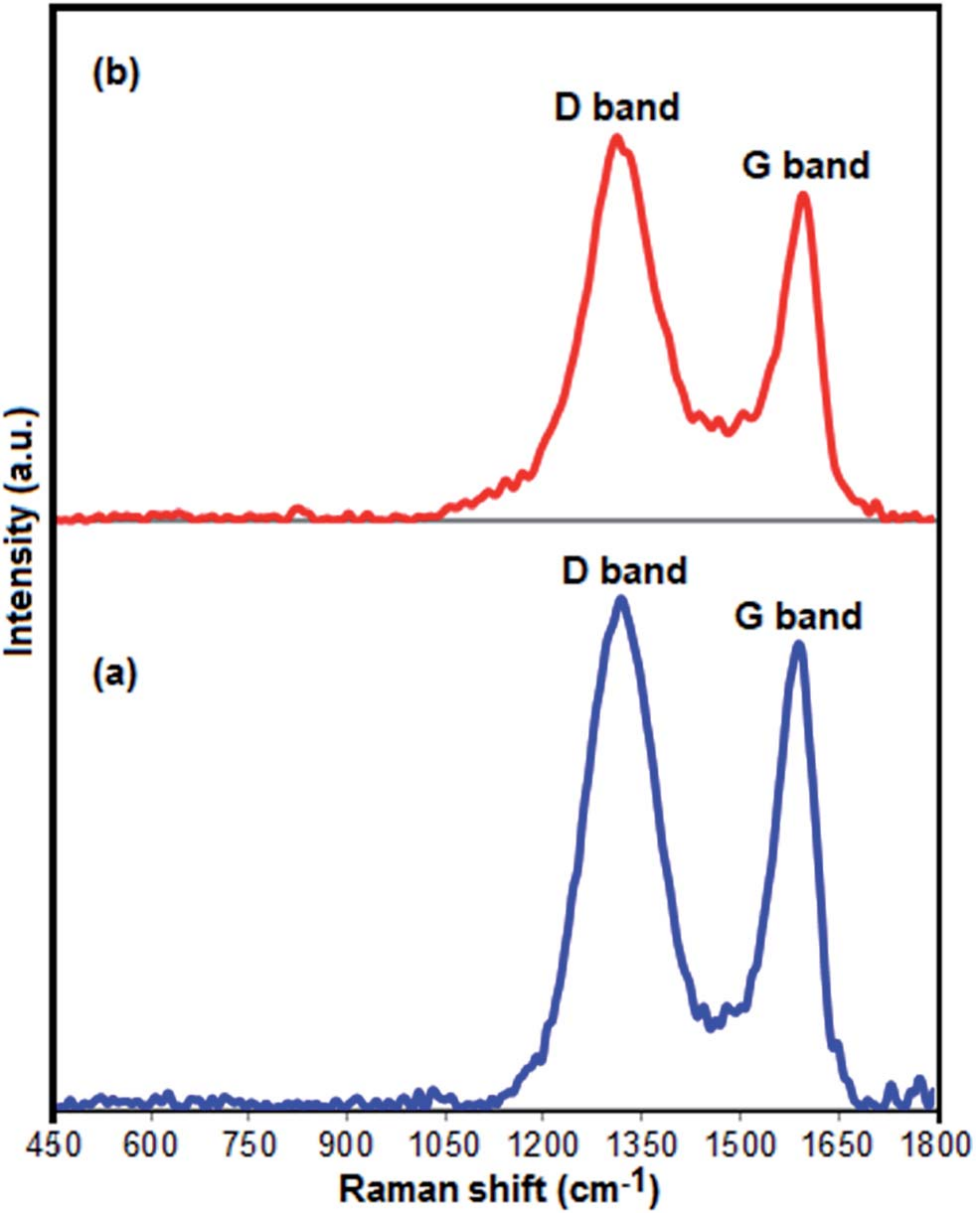
Raman spectra of (a) GO and (b) Fe_3_O_4_/GrOSi(CH_2_)_3_–NH_2_/HPMo.

Fig. 4(a) and (b) presents the Raman spectra of pure GrO and Fe_3_O_4_/GrOSi(CH_2_)_3_–NH_2_/HPMo nanocomposite. Two samples display the characteristic D (sp^3^ carbon atoms of disorders and defects) and G (sp^2^ carbon atoms in graphitic sheets) bands of GrO structure.^66^ As compared with the D and G bands of pure GrO (D, 1318 cm^−1^; G, 1590 cm^−1^), the two characteristic bands of GrO in the Fe_3_O_4_/GrOSi(CH_2_)_3_–NH_2_/HPMo nanocomposite shift to D, 1311 cm^−1^ and G, 1592 cm^−1^, suggesting the successful functionalization of GrO with APTES and HPMo. These shifts in Raman peaks can be attributed to strong interaction between HPMo polyanion and positively charged Fe_3_O_4_/GrOSi(CH_2_)_3_–NH_3_^+^ in the Fe_3_O_4_/GrOSi(CH_2_)_3_–NH_2_/HPMo hybrid,^67^ which is consistent with the FI-IR analysis. Comparing with pristine GrO, the ratio of D and G peaks of Fe_3_O_4_/GrOSi(CH_2_)_3_–NH_2_/HPMo becomes higher, suggesting a higher level of disorder of the graphene layers during the functionalization process.^68^ The peaks of Keggin structure have not been appeared, which indicated that HPMo species on Fe_3_O_4_/GrOSi(CH_2_)_3_–NH_2_ sample were in a high-dispersed state. This result was identical to the XRD results.

**Fig. 5:**
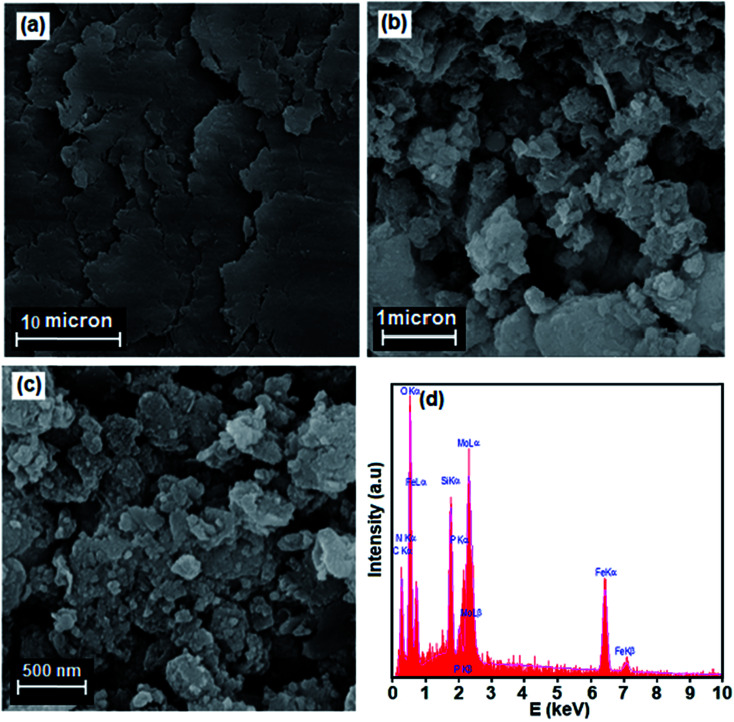
SEM images of (a) GO, (b) and (c) Fe_3_O_4_/GrOSi(CH_2_)_3_–NH_2_/HPMo nanocomposite at different magnification and (d) EDX spectrum of the Fe_3_O_4_/GrO–NH_2_/HPMo nanocomposite.

SEM images indicating the microstructural features of GO and Fe_3_O_4_/GrOSi(CH_2_)_3_–NH_2_/HPMo nanocomposite are shown in Fig. 5. SEM image of pure GO in Fig. 5(a) shows highly porous and layered structure of GO having large stacks, possibly consisting of hundreds of grapheme oxide nanosheets. It should also be noted that the surfaces of the GO sheets are quite flat and smooth. The SEM image of Fe_3_O_4_/GrOSi(CH_2_)_3_–NH_2_/HPMo in Fig. 5(b) and (c) clearly shows graphene oxide nanosheets were successfully decorated with agglomerated Fe_3_O_4_ and HPMo particles that completely covered the surfaces of large graphene sheets. From images, it can be clearly seen that the Fe_3_O_4_ nanoparticles with a size of about 15–20 nm were well deposited on GO nanosheets which were a flexible interleaved structure. Some wrinkles are found on the surface, which may be important for preventing aggregation of GO nanosheets and maintaining high surface area. All the micrographs of the nanocomposite clearly indicated that the surface properties of modified GO product were strongly affected. In opposite of pure GO, the surfaces of GO nanosheets in the nanocomposite are rough, and the edges are highly crumpled. Thus, the Fe_3_O_4_/GrOSi(CH_2_)_3_–NH_2_/HPMo could provide a rough and coarse surface with high porosity for catalytic uses. The results are in good agreement with BET results. The composition of the as-prepared Fe_3_O_4_/GrO–NH_2_/HPMo hybrid nanomaterial was investigated by energy dispersive X-ray spectroscopy (EDX). Fig. 5(d) shows EDX spectrum of the hybrid nanocomposite. The EDX elemental spectrum shows the existence of C, N, Si, O, Fe, P, and Mo elements in the composite. The elements of P and Mo are from HPMo and the results further confirm that the HPMo particles have been successfully supported on the surface of the Fe_3_O_4_/GrOSi(CH_2_)_3_–NH_2_.

**Fig. 6:**
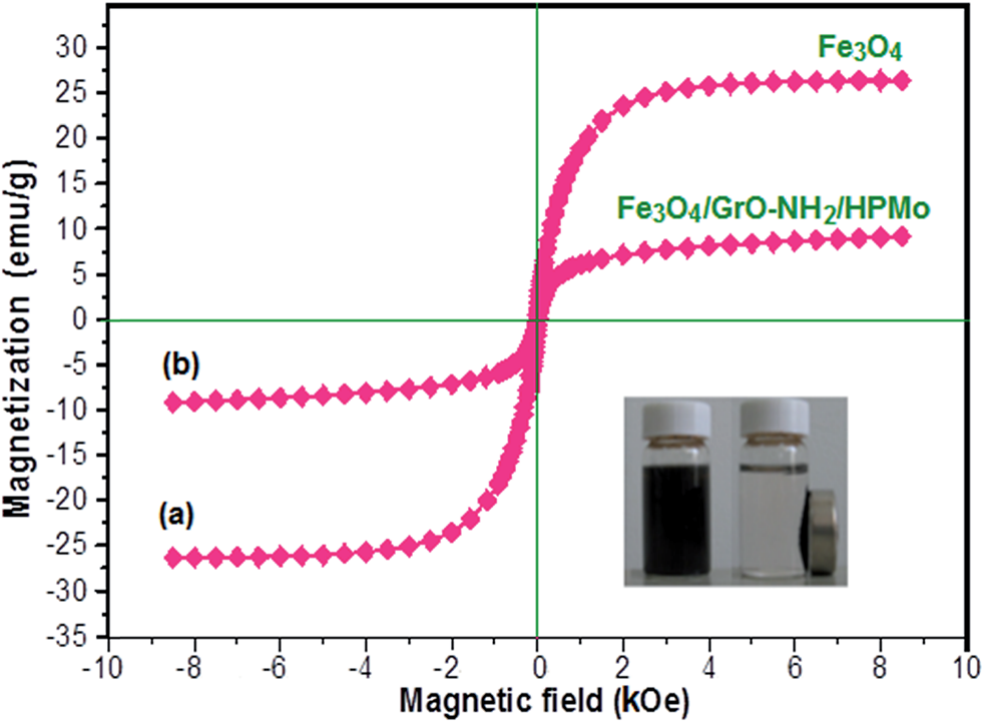
Magnetic hysteresis loop of (a) Fe_3_O_4_ and (b) Fe_3_O_4_/GrOSi(CH_2_)_3_–NH_2_/HPMo at room temperature. The inset shows the behavior of the nanocomposite under an external magnetic field.

The magnetic properties of the Fe_3_O_4_ and Fe_3_O_4_/GrOSi(CH_2_)_3_–NH_2_/HPMo samples were investigated by vibrating sample magnetometry (VSM) at room temperature. The magnetic hysteresis loops are depicted in Fig. 6. It is clear that both Fe_3_O_4_ and the Fe_3_O_4_/GrOSi(CH_2_)_3_–NH_2_/HPMo composite are soft-magnetic materials due to their coercivity (*H*_c_) of zero.^69^ Moreover, they are also superparamagnetic materials, as their magnetic hysteresis loops passed through the origin of the coordinates. The saturation magnetization values of Fe_3_O_4_ and Fe_3_O_4_/GrOSi(CH_2_)_3_–NH_2_/HPMo are 27.50 and 9.15 emu g^−1^, respectively. The saturation magnetization of the magnetic nanocomposite decreases by approximately 75% compared with that of pure Fe_3_O_4_, which can be attributed to the nano-magnetic components (GrO and HPMo) in the composite sample. However, the saturation magnetization of the composite could satisfy the requirements of easy separation in the suspension solution using an extra magnet after reaction as shown in the inset of Fig. 6. Thus, the Fe_3_O_4_/GrOSi(CH_2_)_3_–NH_2_/HPMo composite can be easily separated using a magnetic separation process after being used for the reaction.

**Fig. 7:**
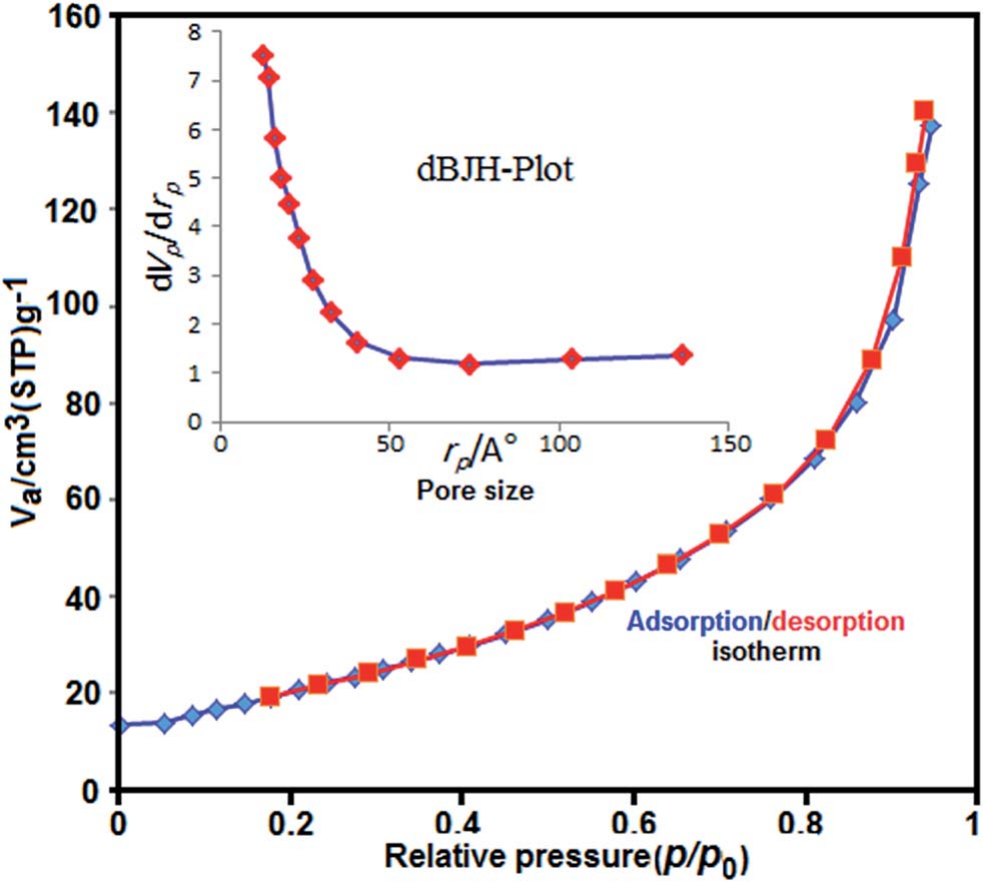
N_2_ adsorption–desorption isotherm of Fe_3_O_4_/GrOSi(CH_2_)_3_–NH_2_/HPMo. The inset shows the pore size distribution plot.

N_2_ adsorption/desorption measurements were performed to investigate specific surface area and the pore size distribution of the Fe_3_O_4_/GrOSi(CH_2_)_3_–NH_2_/HPMo. As can be seen in Fig. 7, the nitrogen adsorption isotherm is a typical IV type curve with an fine H1-type hysteresis loop in the range of *ca.* 0.8–1.0*p*/*p*_0_, indicating the existence of mesoporous structure.^70,71^ The BET surface area is measured to be 76.36 m^2^ g^−1^ that is much higher than the value of pure HPMo (≤10 m^2^ g^−1^).^72^ In addition, the total pore volume is 0.01 cm^3^ g^−1^ and according to the corresponding Barrett–Joyner–Halenda (BJH) pore size distribution curve in the inset of Fig. 7, the pore size distribution of the Fe_3_O_4_/GrOSi(CH_2_)_3_–NH_2_/HPMo shows peak centered at around 2.28 nm. Such porosity of Fe_3_O_4_/GrOSi(CH_2_)_3_–NH_2_/HPMo composite can improve the catalytic performance. It can be concluded that immobilization of HPMo on the surface of magnetic Fe_3_O_4_/GrOSi(CH_2_)_3_–NH_2_ increases its surface area and porosity which they are useful factors for improving the catalytic performance.

### 3.2 Catalytic oxidation of alcohols over Fe_3_O_4_/GrOSi(CH_2_)_3_–NH_2_/HPMo nanocomposite

**Scheme 1:**
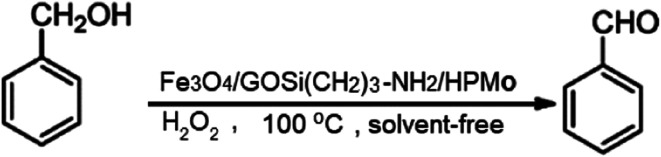


The oxidation of benzyl alcohol to obtain benzaldehyde was used as a typical benchmark reaction to verify the catalytic activity of the as-prepared magnetic Fe_3_O_4_/GrOSi(CH_2_)_3_–NH_2_/HPMo nanocomposite (see Scheme 1). In an initial experiment, a heterogeneous mixture of benzyl alcohol (10 mmol), aqueous H_2_O_2_ (30%, 15 mmol) and a catalytic amount of Fe_3_O_4_/GrOSi(CH_2_)_3_–NH_2_/HPMo (0.2 g) was heated for 4 h at 100 °C, the benzaldehyde was formed as the only product in 90% yield (Scheme 1). In control experiments, when pure Fe_3_O_4_ and GrO support were used as heterogeneous catalysts for the oxidation of benzyl alcohol, the results showed that about 15% and 18% of benzaldehyde was formed, respectively, after 4 h heating at 100 °C under the same reaction conditions, suggesting that the catalytic activity of the Fe_3_O_4_/GrOSi(CH_2_)_3_–NH_2_/HPMo system is mainly due to HPMo heteropoly acid. Using pure and unsupported HPMo, we found that 45% of benzyl alcohol was oxidized to benzaldehyde within 4 h under the same conditions. These findings confirm that the activity of Fe_3_O_4_/GrOSi(CH_2_)_3_–NH_2_/HPMo nanocomposite is higher than that of the pure HPMo cluster. This is due to (i) higher specific surface area of composite than that of the starting POM cluster and (ii) the synergistic effect of the HPMo unit and the GO nanosheets support. No noticeable oxidation products were observed when blank experiments were run in the in the absence of Fe_3_O_4_/GrOSi(CH_2_)_3_–NH_2_/HPMo or H_2_O_2_.

#### 3.2.1 Effect of catalyst dosage

**Fig. 8:**
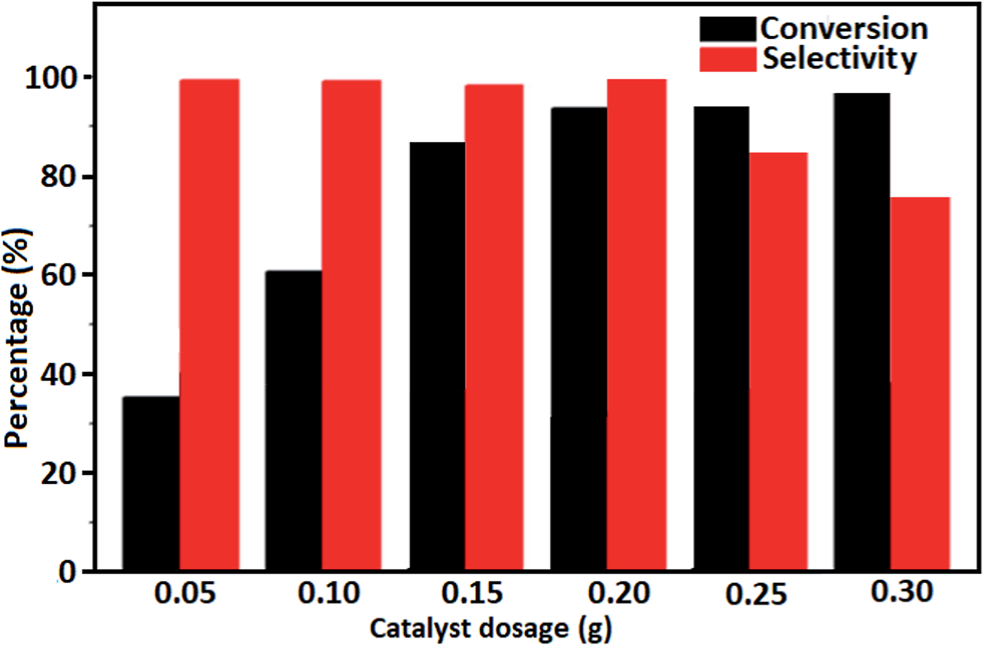
The conversion of benzyl alcohol and the selectivity to benzaldehyde at different catalyst dosage. Conditions: benzyl alcohol (10 mmol), H_2_O_2_ (15 mmol, 30%), catalyst (0.20 g) at reflux temperature 100 °C for 4 h.

The effect of Fe_3_O_4_/GrOSi(CH_2_)_3_–NH_2_/HPMo catalyst dosage on the progress of benzyl alcohol oxidation is illustrated in Fig. 8. With the increase of the catalyst amount from 0.05 g to 0.3 g, the benzyl alcohol conversion increased from 35% to 98%, but the selectivity towards benzaldehyde reduced slightly from 99% to 75%. This may be due to the increase in active sites resulting from higher amount of catalyst which facilitate the further oxidation of benzaldehyde to benzoic acid.

#### 3.2.2 Effect of reaction time

**Fig. 9:**
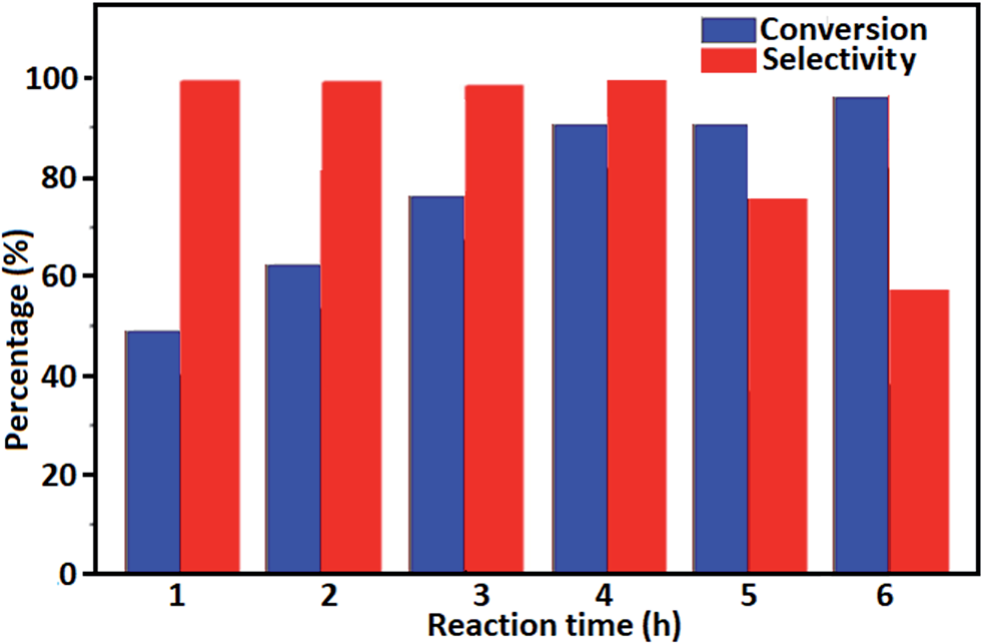
The conversion of benzyl alcohol and the selectivity to benzaldehyde at different reaction time over Fe_3_O_4_/GrOSi(CH_2_)_3_–NH_2_/HPMo as the catalyst. Conditions: benzyl alcohol (10 mmol), H_2_O_2_ (15 mmol, 30%), catalyst (0.20 g) at 100 °C.

Fig. 9 shows the influence of reaction time on the conversion of benzyl alcohol and product selectivity over the Fe_3_O_4_/GrOSi(CH_2_)_3_–NH_2_/HPMo catalyst (0.2 g). As can be seen in Fig. 9, with increase of the reaction time from 1 h to 4 h, the conversion marginally increased from 45% to 90%, and the selectivity towards benzaldehyde remains 100%. However, when the reaction time was increased from 4 h to 5 h and then 6 h, the conversion of benzyl alcohol increased too, but the selectivity towards benzaldehyde declined due to further oxidation of benzaldehyde to benzoic acid. Thus, optimum reaction time was found to be 4 h, when catalyst gave the highest conversion and selectivity, *i.e.* 90% conversion of benzyl alcohol and 100% selectivity towards benzaldehyde.

#### 3.2.3 Effect of oxidant H_2_O_2_ amount

**Fig. 10:**
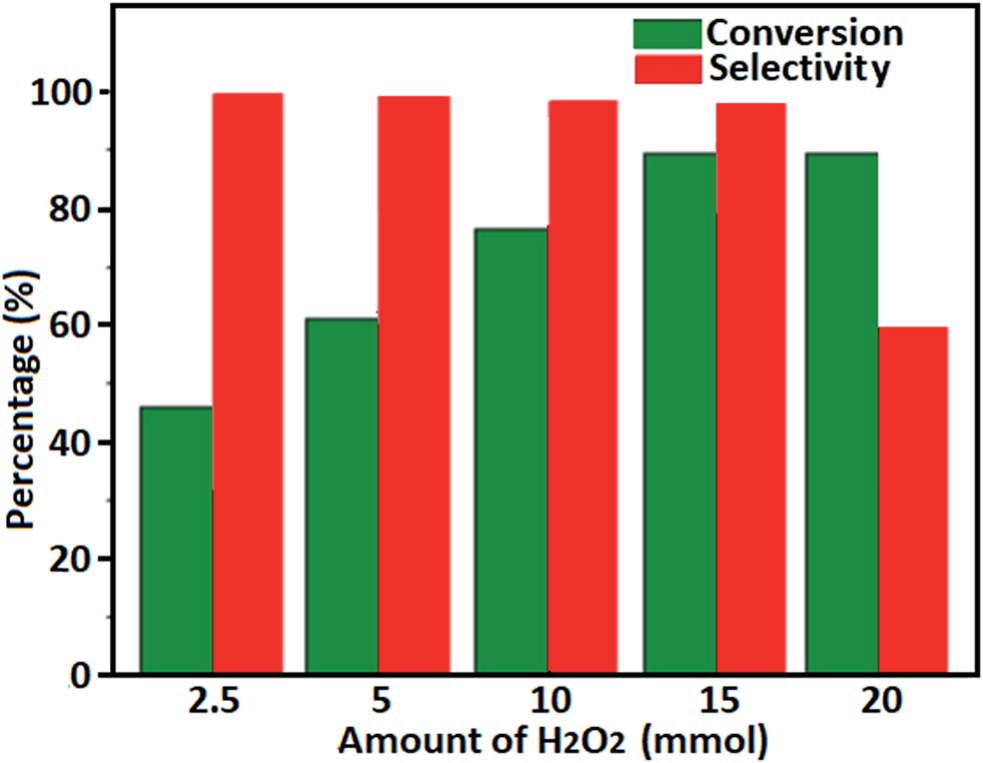
The conversion of benzyl alcohol and the selectivity to benzaldehyde over the Fe_3_O_4_/GrOSi(CH_2_)_3_–NH_2_/HPMo catalyst with different amount of H_2_O_2_ (30%). Conditions: benzyl alcohol (10 mmol), catalyst (0.20 g) at 100 °C for 4 h.

The amount of hydrogen peroxide is another important parameter influencing the results of this oxidation reaction. According to stoichiometry, one mole of H_2_O_2_ is required to convert one mole of benzyl alcohol to one mole of benzaldehyde. Effect of mole ratio of H_2_O_2_/benzyl alcohol was investigated in range of 1.0–2.0 and the results are given in Fig. 10, from which a lower conversion of benzyl alcohol was observed with 1.0 mole ratio of H_2_O_2_/benzyl alcohol. Although a higher conversion of benzyl alcohol was obtained when the mole ratio of H_2_O_2_/benzyl alcohol is 2.0, the percentage selectivity to benzaldehyde was reduced around 40%, resulted by the excess unreacted H_2_O_2_ in reaction mixture, as compared to that obtained with 1.5 mole ratio of H_2_O_2_ to benzyl alcohol. Based on the results, the mole ratio of 1.5 was chosen as the optimal ratio of H_2_O_2_/benzyl alcohol.

#### 3.2.4 Effect of temperature

**Fig. 11:**
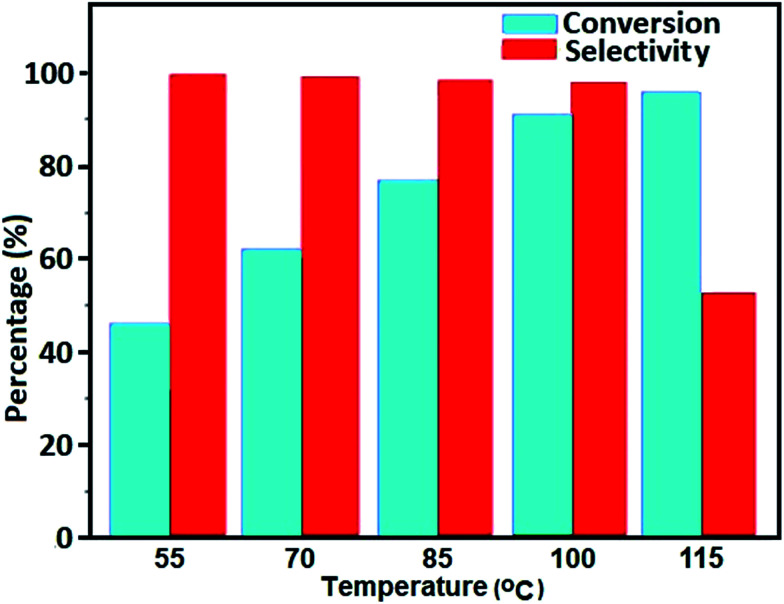
Effect of temperature on the conversion of benzyl alcohol using the Fe_3_O_4_/GrOSi(CH_2_)_3_–NH_2_/HPMo catalyst. Conditions: benzyl alcohol (10 mmol), H_2_O_2_ (15 mmol, 30%), catalyst (0.20 g) at 100 °C for 4 h.

The oxidation reaction was investigated at five temperatures, 55, 70, 85, 100 and 115 °C, while other parameters are kept fixed (Fig. 11). The conversion% increased with increasing temperature from 55 to 115 °C, but the selectivity towards benzaldehyde drastically decreased at 115 °C. This might be due to the self-decomposition of H_2_O_2_ at higher temperatures and/or further oxidation of benzaldehyde to benzoic acid at elevated temperatures.

Thus, optimized conditions for oxidation of benzyl alcohol (90% conversion, 100% selectivity of benzaldehyde) as a model reaction are as follows: amount of the catalyst (0.2 g), temperature (100 °C), H_2_O_2_ oxidant to alcohol ratio (1.5) and reaction time (4 h).

#### 3.2.5 Oxidation of various alcohols

Under the optimized reaction conditions for oxidation of benzyl alcohol, we explored the substrate scope of this catalytic oxidation system. The oxidation of a series of primary and secondary benzylic alcohols with H_2_O_2_ in the presence of Fe_3_O_4_/GrOSi(CH_2_)_3_–NH_2_/HPMo catalyst was studied. We found that these substrates are efficiently oxidized to the corresponding carbonyl compounds in high yield without observable over-oxidation of the benzaldehydes to the carboxylic acids. The results were presented in Table 1. As can be seen in Table 1, H_2_O_2_-mediated oxidation a variety of ring-substituted benzyl alcohols having various electron-withdrawing and -donating substituents *e.g.* iso-Pr–, –OMe, –NO_2_, and halogens (–Br and –Cl) was investigated over the Fe_3_O_4_/GrOSi(CH_2_)_3_–NH_2_/HPMo catalyst. The results showed that all primary benzyl alcohols were selectively converted to their corresponding aldehydes in moderate to high yields without over-oxidation to the carboxylic acids (Table 1, entries 1–10). The yields were found to be affected by the substituent groups on the phenyl ring of benzylic alcohols somewhat. Generally, alcohols having stronger electron withdrawing groups gave products with lower yields compared with those having electron donating groups on the phenyl rings and benzyl alcohol (Table 1, entries 1–6). These results indicated that electron density on the aromatic ring played an important role in the reactivity of the oxidation reaction. On the other hand, attachment of halogen groups to the *para*-position of the aromatic ring increased the efficiency of the oxidation reaction (Table 1, entries 7 and 8) while *ortho*-substituted derivative gave lower yield (Table 1, entries 9). The slightly improvement of yield in 4-Br- and 4-Cl-benzyl alcohols is probably due to positive resonance effect (+R) of halide groups at *para*-position, activating of the benzene ring. Steric hindrance is another important factor that affects the reactivity as electron withdrawing –NO_2_ and –Cl groups attached to the aromatic ring at *ortho*-position decreased the efficiency of the reaction. In addition, a 2,4-dichloro substitution did not reduce or increase the yield (Table 1, entry 10), confirming that the catalytic performance is dependent on the electronic (inductive and resonance) effect and steric effect of the substituents present on the phenyl ring. From the above findings, it is clear that an extended pi structure conjugated with the aldehyde and aromatic rings with high electron density increase the yield. Various secondary benzylic alcohols were also converted with high selectivity (≥99%) to their corresponding ketones without any side reactions (Table 1, entries 11–13). Cinnamyl alcohol, as an allylic alcohol, was selectively converted into the corresponding unsaturated aldehyde in high yield without oxidation of carbon–carbon double bond (Table 1; entry 14). In contrast to benzylic and allylic alcohols which were oxidized in a highly efficient way, non-benzylic alcohols such as and 3-phenyl-1-propanol, 1-heptanol, and 2-octanol were oxidized into the corresponding carbonyl compounds with much lower efficiency under the same reaction conditions (Table 1; entries 15–17). Also, alicyclic alcohol such as cyclohexanol was selectively oxidized to the corresponding cyclic ketone in moderate yield (Table 1; entry 18). The GC yields of these conversions were in the range of 60–68% after reaction time of 4 h. From the above findings, it is clear that an extended pi structure conjugated with the aldehyde increase the yield.

**Table 1:** Results of various alcohol oxidation with H_2_O_2_ catalyzed by the Fe_3_O_4_/GrOSi(CH_2_)_3_–NH_2_/HPMo catalyst^a^

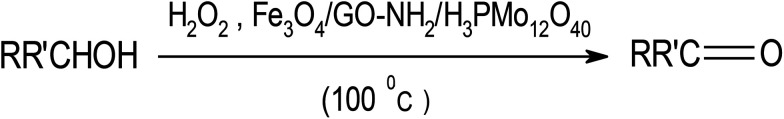			
Entry	Substrate	Product	Yield^b^ (%)
1	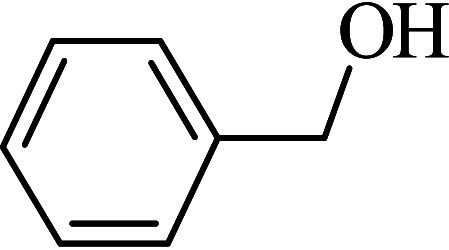	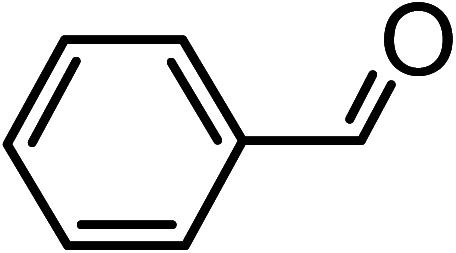	90
2	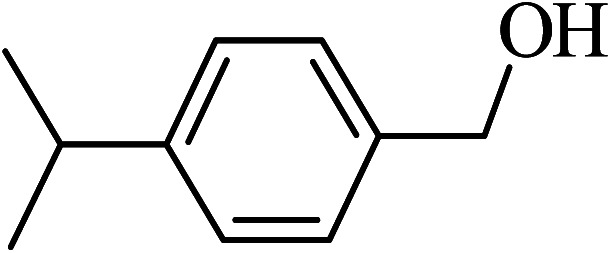	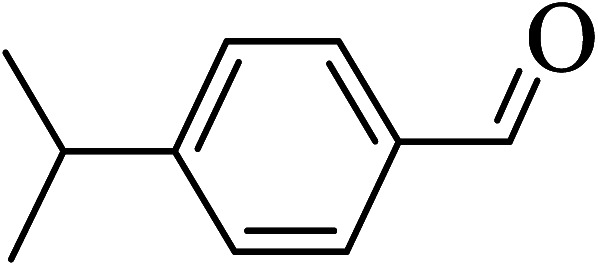	96
3	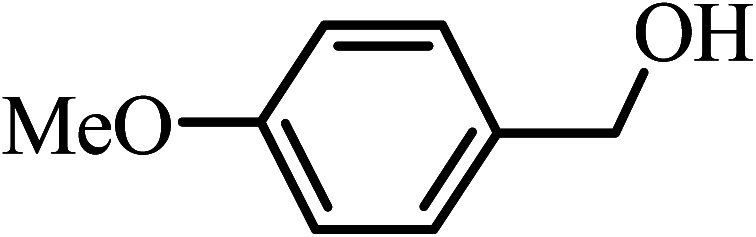	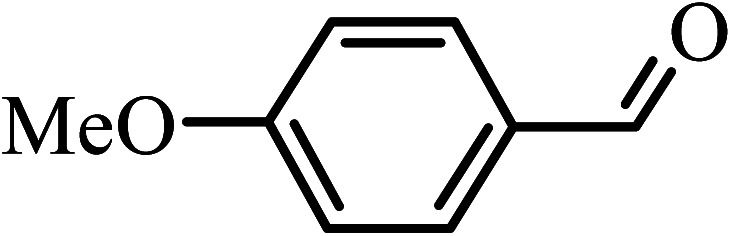	94
4	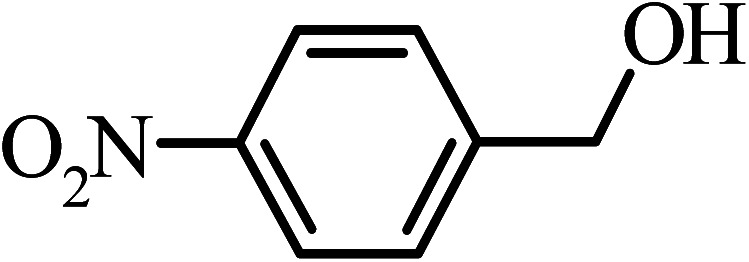	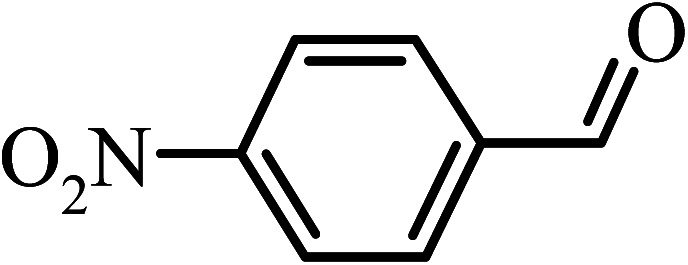	86
5	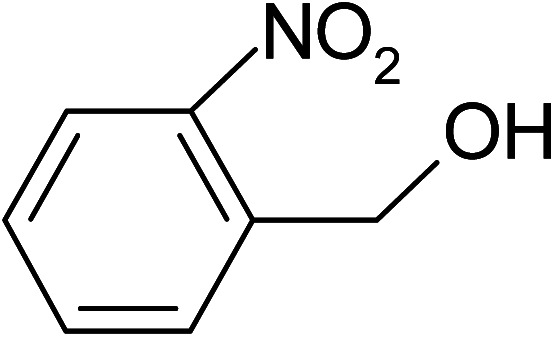	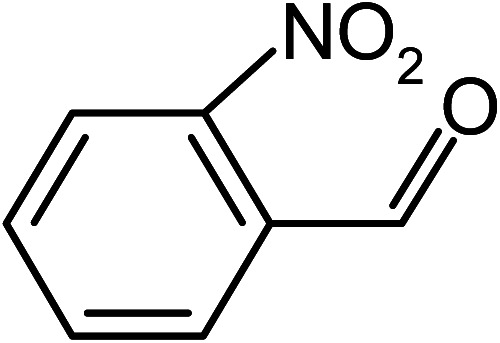	60
6	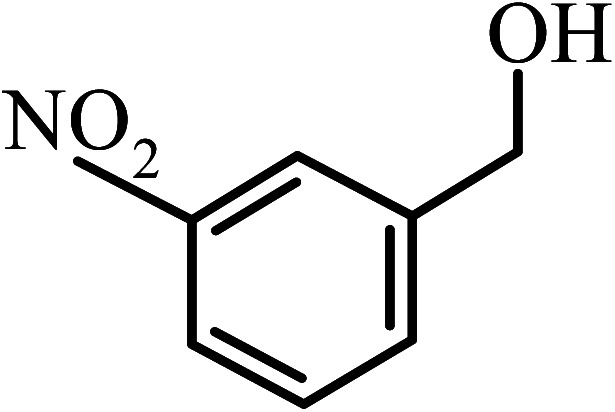	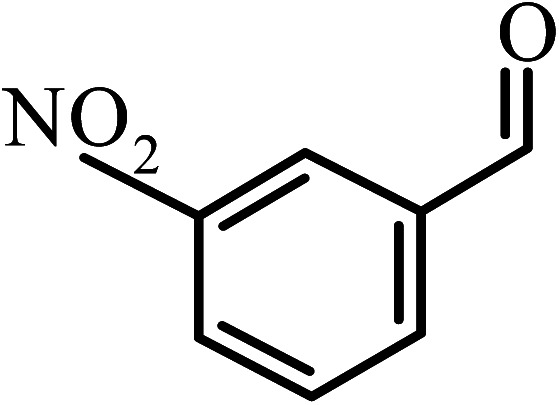	82
7	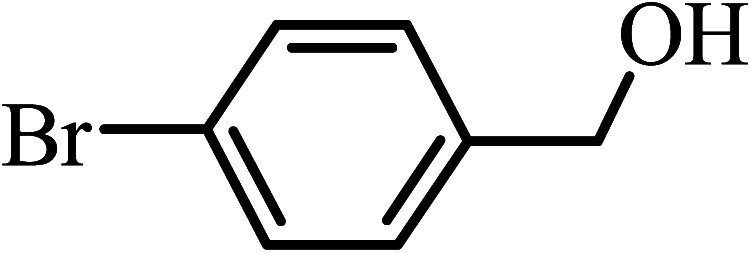	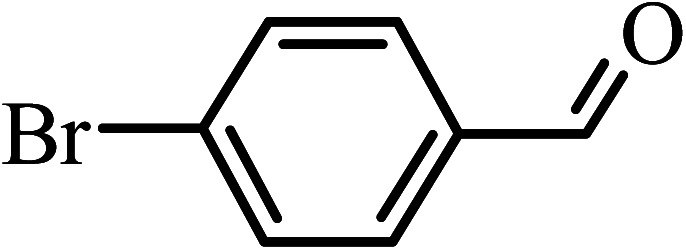	92
8	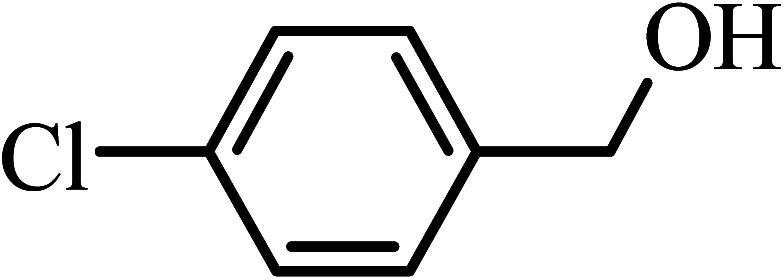	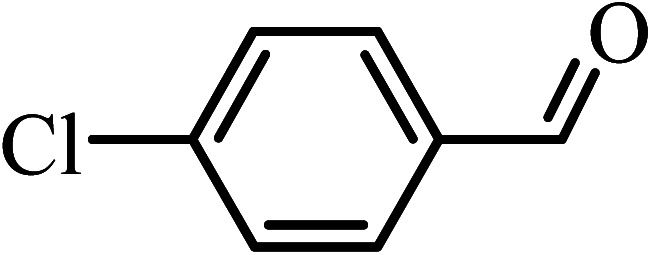	94
9	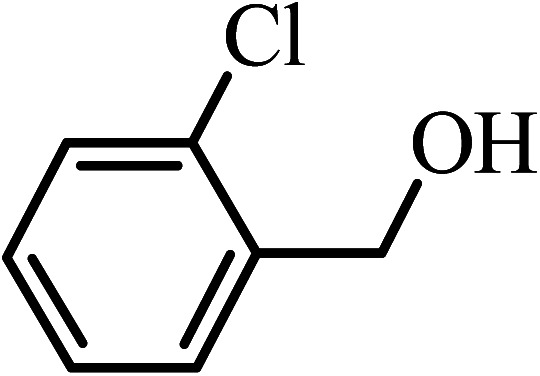	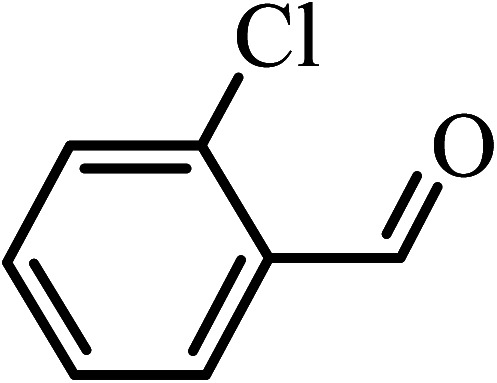	82
10	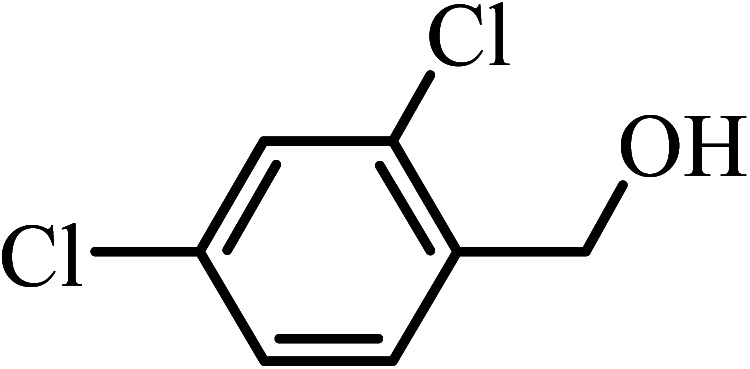	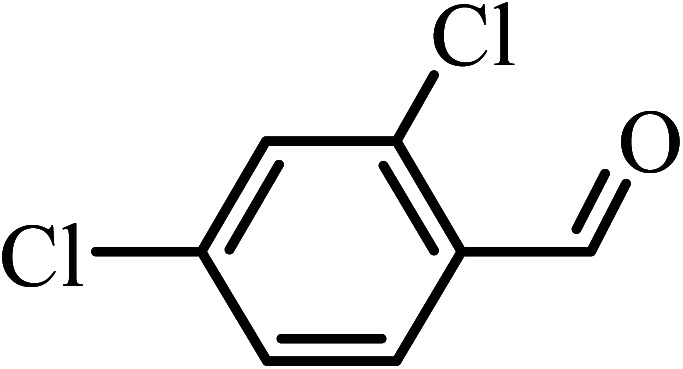	90
11	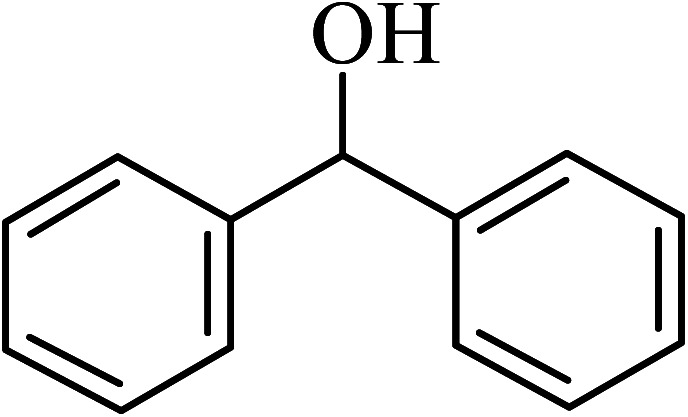	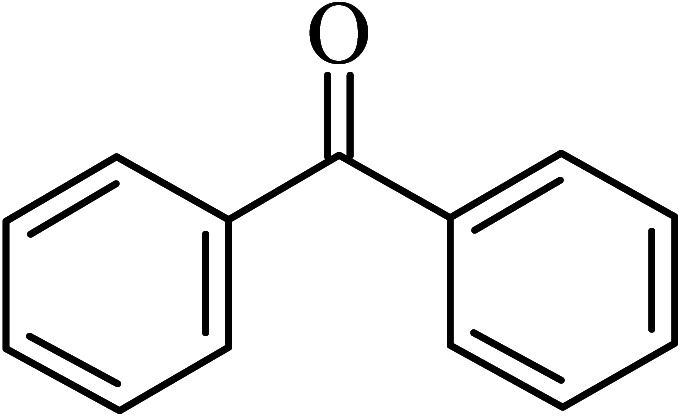	90
12	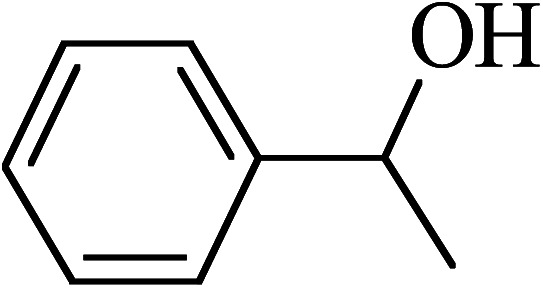	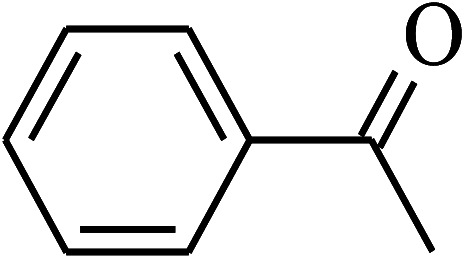	90
13	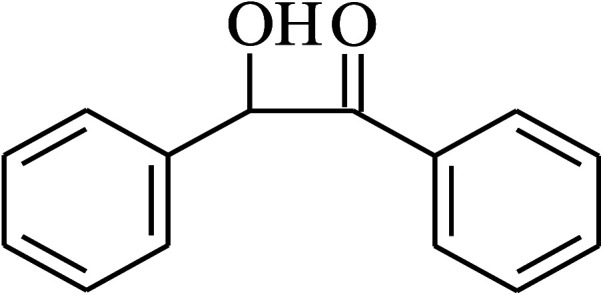	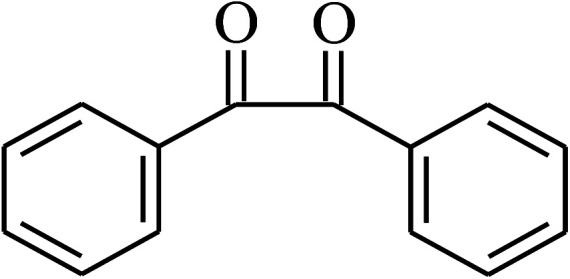	90
14	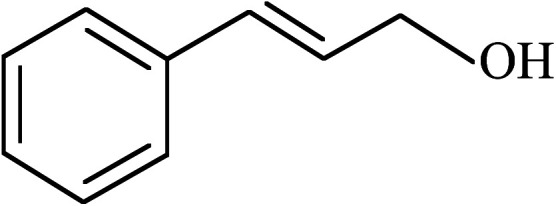	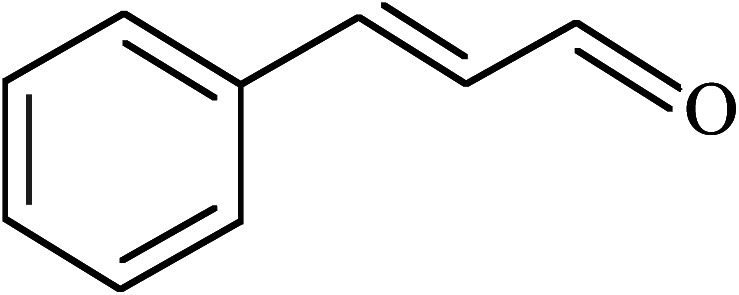	88
15	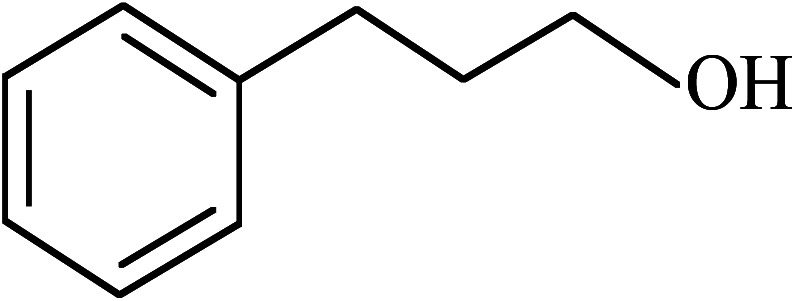	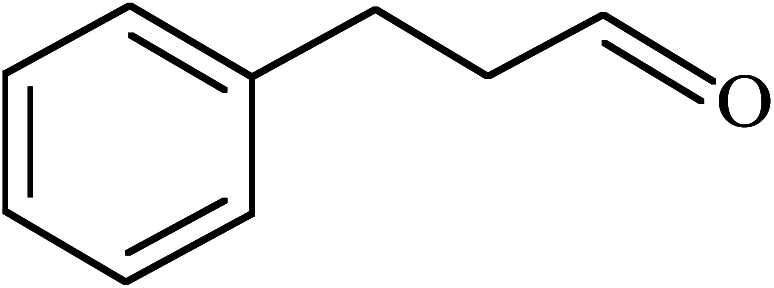	65
16	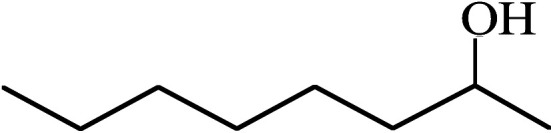	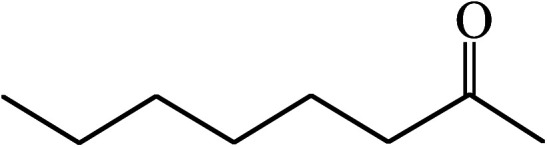	65
17	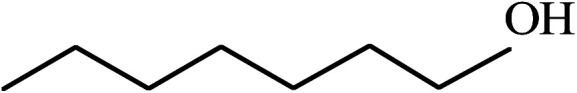	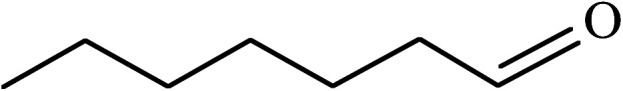	60
18	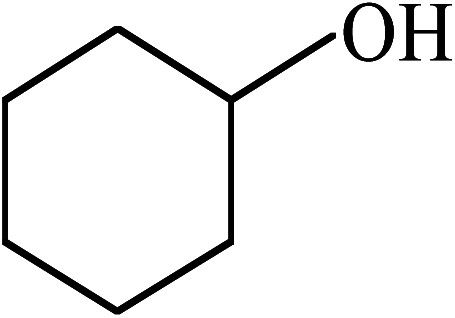	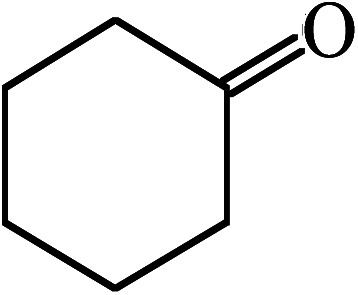	68

a Reaction conditions: alcohol (10 mmol), catalyst (0.2 g), H_2_O_2_ (15 mmol, 30 wt%), 100 °C under solvent-free conditions for 4 h.

b Yields were determined by GC analysis with *n*-decane as internal standard.

The experiments, usually performed on a 10 mmol scale, can be scaled up to 100 mmol without difficulties. A 50 mmol reaction of 4-methoxybenzyl alcohol provided the corresponding aldehyde in a 92% yield and a 100 mmol reaction of 1-phenylethanol gave acetophenone in an 88% yield.

#### 3.2.6 Stability and reusability of the catalyst

**Fig. 12:**
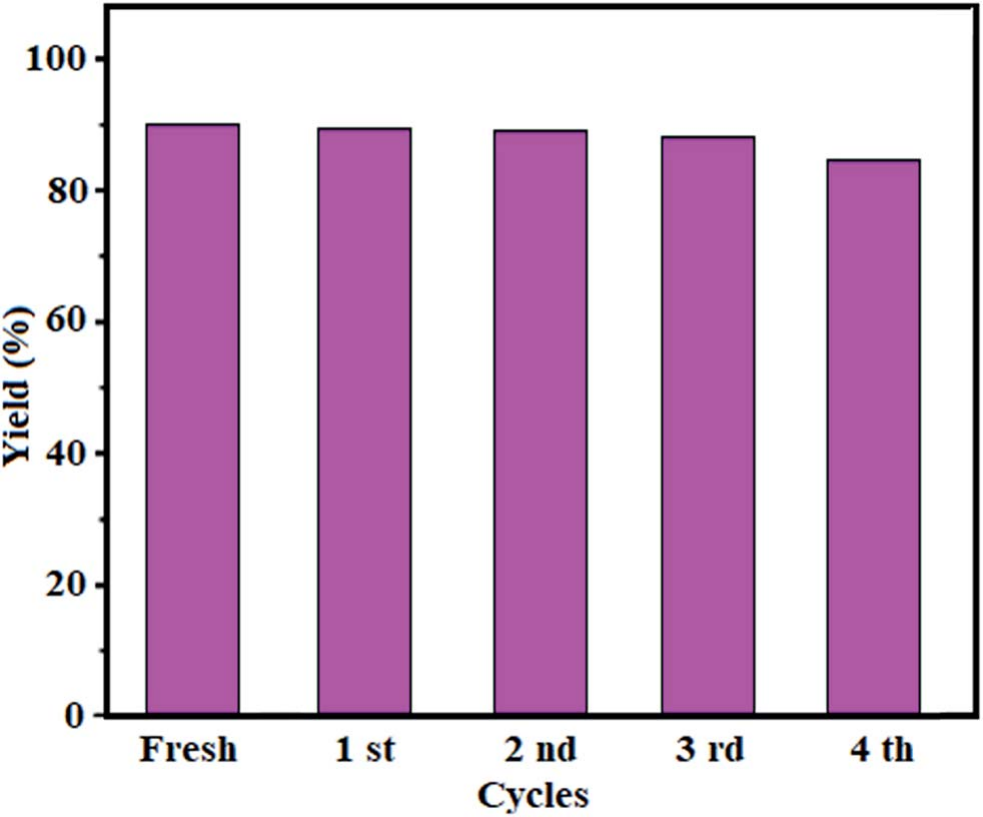
Recyclability of the Fe_3_O_4_/GrOSi(CH_2_)_3_–NH_2_/HPMo catalyst. Conditions: benzyl alcohol (10 mmol), H_2_O_2_ (15 mmol, 30%), catalyst (0.20 g) at 100 °C for 4 h. Yields are for isolated pure benzaldehyde.

Stability and reusability of the catalysts are very important for commercial applications. The reusability of Fe_3_O_4_/GrOSi(CH_2_)_3_–NH_2_/HPMo catalyst in the selective oxidation of benzyl alcohol to benzaldehyde with 30% H_2_O_2_ was investigated, the results were summarized in Fig. 12. After completion of the reaction, the catalyst was easily recovered from the reaction mixture by an external magnet and then used for the subsequent catalytic runs without further activation. Interestingly, the catalyst could be quantitatively recovered from the reaction mixture and reused for several times without significant loss in conversion and selectivity (Fig. 12). As is shown in Fig. 12, a slight decrease in the yield of benzaldehyde from 90% with fresh catalyst to about 87% in the fourth run was observed. The molybdenum content in aqueous phase after reaction was analyzed by ICP-AES. On the other hand, no detectable leaching of Mo was observed in the first as well as the four run of the reaction. Also, in an experiment when the catalyst separated from the reaction mixture shortly (1 h) after the beginning the reaction and the filtrate was further heated under our reaction condition, no extra formation of benzaldehyde was observed *via* GC analysis even after 4 h and the oxidation was completely stopped by the removal of the catalyst. These observations confirm that the reaction catalyzed by the Fe_3_O_4_/GrOSi(CH_2_)_3_–NH_2_/HPMo nanocomposite is heterogeneous in nature.

**Fig. 13:**
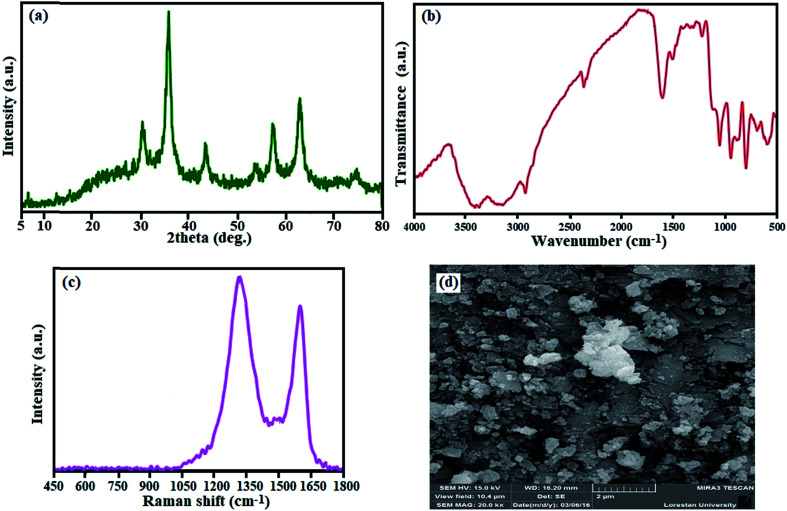
(a) XRD pattern, (b) FT-IR spectrum, (c) Raman spectrum and (d) SEM image of the recovered Fe_3_O_4_/GrOSi(CH_2_)_3_–NH_2_/HPMo nanocomposite after fourth run.

The stability of this material is further discussed. As shown in Fig. 13(a)–(c), the XRD pattern, FTIR and Raman spectrum of the recovered catalyst after fourth run are consistent with those of the fresh catalyst (see Fig. 2(b), 3(b) and 4(b)). These observations confirm that the structure of the Fe_3_O_4_/GrOSi(CH_2_)_3_–NH_2_/HPMo catalyst is stable under the reaction conditions and is not affected by the reactants. The morphology of the recycled catalyst particles was also analyzed. Fig. 13(d) shows catalyst almost kept initial size and morphology even after four runs (see Fig. 5(b) and (c)). The surface of GO nanosheets is still decorated with spherical Fe_3_O_4_ and white HPMo particles revealing the strong binding between the Fe_3_O_4_ nanoparticles and HPMo with amino functionalized GO nanosheets. Considering the above mentioned experiment results, we can conclude that the structure of the catalyst remains intact, which further confirms its excellent stability and recyclability.

## 4. Conclusion

In conclusion, the Fe_3_O_4_/GrOSi(CH_2_)_3_–NH_2_/HPMo nanocomposite was prepared and tested as a novel magnetically separable oxidation catalyst under solvent free conditions. Selective oxidation of benzyl alcohol to benzaldehyde was used as a benchmark reaction to evaluate the catalytic performance of the as-prepared catalyst. Under the optimized conditions, H_2_O_2_-oxidation of a wide range of alcoholic substrates gave the desired carbonyl compounds in moderate to excellent yields. This reaction is highly selective (≥99%) for benzylic alcohols. In the oxidation of benzylic alcohols, strong electron-withdrawing substituents induced lower yields than others. This indicates that carbocation-type intermediates are involved in the catalytic oxidation reaction due to hydride elimination from the beta proton of substrate. In addition, it is easy to separate by an external magnet and reuse the catalyst for another catalytic recycling. The catalytic performances of the re-used catalyst, even for using four times, were comparable with that of the fresh catalyst, giving this catalyst a good prospect for benzaldehyde production in industry. Also, the present work provides a new type of heterogeneous catalytic materials for selective organic transformations. Work on the detailed mechanism of this catalytic reaction is under investigation in our laboratory.

## Conflicts of interest

There are no conflicts of interest to declare.

## Supplementary Material
